# Suppurative Stomatitis [Cheilitis Glandularis]: an underreported entity

**DOI:** 10.4317/medoral.27138

**Published:** 2025-08-16

**Authors:** Juan Cruz Romero Panico, Bruno Augusto Benevenuto de Andrade, Jerónimo Lazos, Rocío Martin, Eduardo Piemonte, Caio Almeida Moreira Lopes, Clara Herrera Freire, René Luis Panico, Gerardo Gilligan

**Affiliations:** 1 ORCID: 0009-0001-7263-1886. Oral Medicine Department, Dentistry Faculty, National University of Cordoba, Argentina; 2 ORCID: 0000-0002-3259-606X. Oral Diagnosis Department, Faculty of Dentistry, Federal University of Rio de Janeiro, Brazil; 3 ORCID: 0000-0002-0881-9614. Oral Medicine Department, Dentistry Faculty, National University of Cordoba, Argentina; 4 No ORCID Number. Oral Medicine Department, Dentistry Faculty, National University of Cordoba, Argentina; 5 ORCID: 0000-0002-5499-3568. Oral Diagnosis Department, Faculty of Dentistry, Federal University of Rio de Janeiro, Brazil; 6 ORCID: 0000-0002-9757-5577. Oral Diagnosis Department, Faculty of Dentistry, Federal University of Rio de Janeiro, Brazil; 7 ORCID: 0000-0001-5955-1139. Oral Medicine Department, Dentistry Faculty, National University of Cordoba, Argentina; 8 ORCID: 0000-0002-5833-5546. Oral Medicine Department, Dentistry Faculty, National University of Cordoba, Argentina; 9 ORCID: 0000-0002-5201-1444. Oral Medicine Department, Dentistry Faculty, National University of Cordoba, Argentina

## Abstract

**Background:**

Stomatitis and Cheilitis Glandularis are inflammatory conditions of the minor salivary glands with uncertain aetiology, uncommon in clinical practice. Local factors and systemic comorbidities associated with hyposalivation could contribute to the onset of the disease. The aim of this study is to describe retrospectively a case series of Stomatitis and Cheilitis Glandularis from two Oral Medicine and Pathology Departments. Also, a review of the clinical and epidemiological variables and a discussion of the diagnostic criteria is presented.

**Material and Methods:**

There were included cases of Stomatitis and Cheilitis Glandularis from two Oral Diagnosis Centres from Argentina and Brazil, according to Reiter *et al* criteria. Demographic and clinicopathological features were described. A concise review of current literature was performed discussing diagnostic criteria and possible etiological factors.

**Results:**

There were included six cases sequentially retrieved from the last ten years. The patients included were 4 men and 2 women with an average age of 65.33 years old. The majority of the included cases showed comorbidities or were treated with drugs associated with hyposalivation or a decrease in salivary flow. Clinically, firm nodules and pus discharge was observed in minor salivary glands. Actinic damage was a poorly associated factor in this series. Histopathology revealed sialoadenitis, oncocytic metaplasia with different degrees of periductal inflammation in all cases.

**Conclusions:**

Stomatitis and Cheilitis Glandularis are challenging disorders of the oral cavity. Hyposalivation could be an early phenomenon in a multifactorial context. More studies are needed for a better understanding of these conditions.

** Key words:**Stomatitis glandularis, cheilitis glandularis, minor salivary glands.

## Introduction

Suppurative Stomatitis Glandularis (SSG) is a chronic inflammatory disease of the minor salivary glands with uncertain aetiology. It is characterized by a chronic inflammatory process with deep micro abscesses formation that drain through the excretory ducts (1,2). It was previously described as Stomatitis Glandularis by Williams & Williams as an evolution of persistent sialadenitis comprising minor salivary glands of the buccal mucosa and palate. Cheilitis Glandularis (CG) is considered a particular entity, encompassing minor salivary glands of the lips (3). CG is limited to the lips and is more common than the larger salivary gland compromise seen in SSG. Clinically, CG shows mild reddening of the lower lip vermilion, in initial cases, up to evident suppuration from the salivary ducts (4). CGs are considered part of the broad spectrum of pathological conditions that can affect the lips (cheilitis), which are a frequent reason for consultation with dermatologists or oral medicine specialists (5).

SSG and CG are the same entity but with different anatomical involvement of minor salivary glands and they could share a similar epidemiological profile and aetiology, with a predominance for adult males (3). However, in CG exogenous factors such as actinic radiation, and dry and windy weather are the most relevant risk factors. Local factors, such as poor oral condition, chronic tobacco consumption and immunosuppression have also been mentioned with CG (1). Piperi *et al*. hypothesized that initially, CG can display hyposalivation and qualitative alterations in saliva, increasing its viscosity. Subsequently, several external factors could worsen the condition, with more inflammation and retrograde infection, causing mucopurulent discharge (4).

The histological criteria mention sialectasia, chronic inflammation, oncocytic or mucosal metaplasia in ducts or acini, and the presence of ductal mucins (6). Two or more of such findings should be present for diagnosis (4). A recent published systematic review included 57 cases of CG from 40 articles that met the inclusion criteria, with 68.4% of cases occurring in males and 31.6% in females, with a mean age of 40.9 years. Clinical manifestations ranged from asymptomatic cases to discomfort, pain, swelling, erythema, lip eversion, dilated ductal openings, ulcers, and crusts. The lower lip was exclusively affected in 71.9% of cases, the upper lip in 7.0%, and both lips in 21.1%. Treatment varied, with surgical intervention being the most common (42.1%), while conservative approaches with topical medications led to resolution in 21.0% of cases (7). However, the involvement of extralabial minor salivary glands, leading to a SSG diagnosis, is not clearly described.

The aim of this study is to describe retrospectively a case series of SSG and CG from two Oral Medicine and Pathology Departments.

## Material and Methods

A retrospective descriptive study was conducted on cases diagnosed at two Oral Diagnostic Centers located in Argentina and Brazil. Cases consistent with SSG and CG were retrieved from the digital registries and databases of both centers. For the inclusion of selected cases, the criteria established by Reiter *et al*. (6), addressing clinical (multiple lesions: involvement of more than one minor salivary gland, and the presence of mucoid and/or purulent discharge from the apertures of the involved minor salivary glands) and histopathological features (biopsies revealing sialectasia, chronic inflammation, mucous/oncocytic metaplasia in ducts or acini, and the presence of mucin in ducts), were applied for the diagnosis of CG and SSG. Cases from the last ten years were included in the analysis. Clinicopathological characteristics were recorded, and all diagnoses were confirmed histopathologically. Cases lacking complete data on relevant inclusion variables were excluded from this study.

Ethical approval: All procedures performed in studies involving human participants adhered to the ethical standards of the 1964 Helsinki Declaration and its later amendments or comparable ethical guidelines. This study was approved by the Academic Committee of Science Research (Comité Académico de Investigación en Ciencias de la Salud) of the Facultad de Odontología, Universidad Nacional de Córdoba, Argentina. Informed consent was obtained from all individual participants included in the study.

Table 1 presents a summary of the main demographic, clinical, and histopathological characteristics of the analyzed cases.

**Figure 1:**
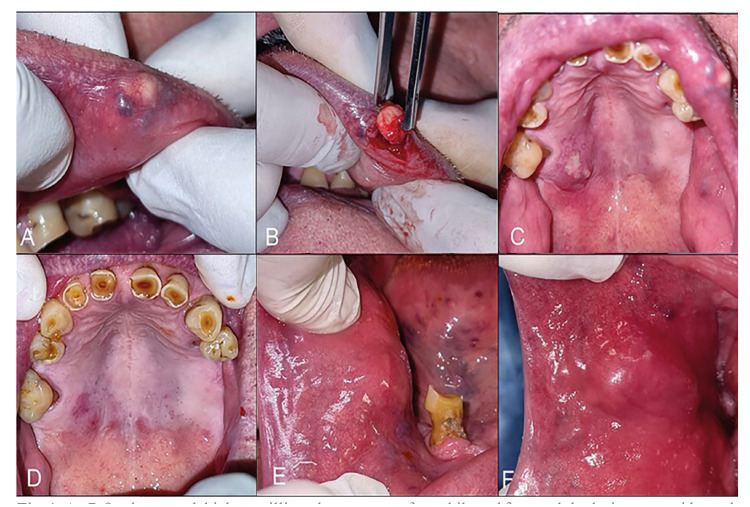
A - B On the upper labial vermillion, the presence of a mobile and firm nodular lesion was evidenced, having consistency compatible with sialolithiasis. The labial nodule was easily removed. Histopathological findings were consistent with minor salivary glands sialolith. C-D: Pus discharge from several palatal salivary glands and its evolution after antibiotics therapy (Amoxicillin/clavulanic acid (875/125 mg) plus metronidazole 250 mg was indicated for two weeks, with substantial clinical improvement).E-F: Swollen nodule with pus discharge on the right buccal mucosa (first consultation); same site after two weeks of antibiotic treatment and dental extraction. The lesion showed limited improvement after antibiotics leading to a biopsy to confirm diagnosis. This case showcase multiple intraoral involving of minor salivary glands.

Case 1: A 78-year-old male patient was referred by his dentist with several intraoral painful nodular lesions, causing discomfort during eating with evolution of 2 months. The patient was a former smoker, he was under treatment for hypertension (propranolol). He related a history of depression, taking clonazepam 0.5mg/day and sertraline 25mg. No other relevant history was recorded. On the buccal mucosa, erythematous and firm nodules were found, with a smooth surface and showing multiple fistulas. On the palatal fibromucosa, a painful swelling involving salivary glands was observed. All lesions, including the palatal lesion, showed purulent discharge. On the upper labial vermillion, a firm nodular lesion was also evidenced, having consistency compatible with sialolithiasis. Also, halitosis, ill-fitting dentures, and poor hygiene were evidenced (Fig. 1). The laboratory analysis showed no significant alterations. No specific germs were isolated in the microbial culture obtained from the purulent discharge. Amoxicillin/clavulanic acid (875/125 mg) plus metronidazole 250 mg was indicated for two weeks, with significant clinical improvement.

A biopsy of a nodule on the right buccal mucosa showed areas of chronic sialadenitis, with periductal inflammation and oncocytic metaplasia in the glandular ducts. In this context, SSG was diagnosed. The nodule located on the upper labial vermillion was also excised, corroborating the presence of a sialolith. The patient showed no recurrence of oral lesions in the follow-up after four months.

**Figure 2:**
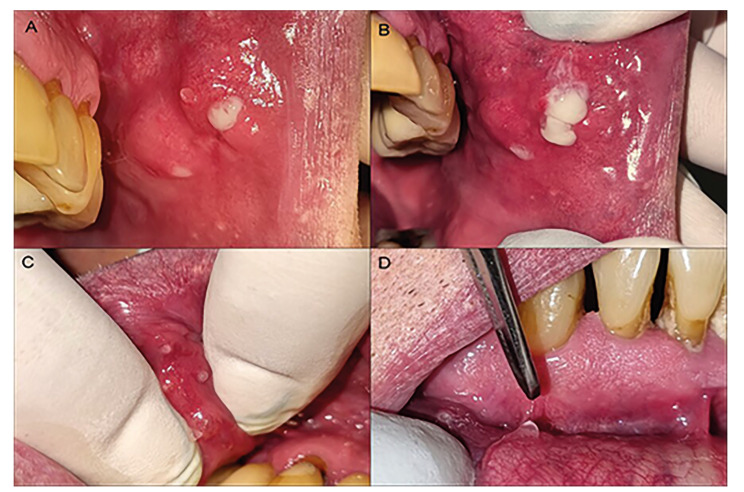
A-B-C Openings of the minor salivary glands inflamed and dilated, and pressure produces mucopurulent secretions (buccal and labial mucosa); D) Thick and slimy saliva out of a lip gland.

Case 2: A 71-year-old male patient was referred with intraoral nodular lesions of 3 years of evolution. The lesions were painless but the patient described a feeling of “the lips compressed”. He suffered from chronic gastritis, and depression and was treated with clonazepam 0.5mg/day. He was also a two-pack-a-day smoker for the past 30 years. On oral inspection, two nodular lesions located on the upper labial and buccal mucosa were observed with pus discharge on palpation. On the lower labial mucosa, hypertrophy and inflammation of the glands were observed with tick saliva, and pressure on the glands producing mucopurulent secretion (Fig. 2). The biochemical profile showed normal values, ruling out diabetes. No specific germs were isolated in the culture from the collected purulent material, except for the presence of gram-positive cocci. Amoxicillin 875 mg with clavulanic acid 125 mg and metronidazole 250 mg was prescribed for two weeks. A nodule that was still suppurating was biopsied, revealing areas of chronic sialadenitis, slight periductal inflammation, and oncocytic metaplasia in the glandular ducts. Because of the specific involvement of lip salivary glands, a diagnosis of CG was made. The condition undergoes partial improvement, with no purulent discharge.

Case 3: A 75-year-old female patient was referred with a complaint of an asymptomatic indurated lip swelling associated with mucopurulent discharge on the lower lip of three months. The patient had a history of Sjogren syndrome under treatment. She did not present chronic mechanical irritation of oral mucosa, solar exposure and denied alcohol or smoke consumption. The clinical impression was of a mucocele or CG, and an excisional biopsy was performed. Histopathological findings consisted of an intense inflammatory mononuclear infiltrate associated with ductal ectasia and enlarged salivary ducts with oncocytic metaplasia. Based on that, a diagnosis of CG was made. The patient is under regular monitoring and no signs of recurrence were observed after 2 years.

**Figure 3:**
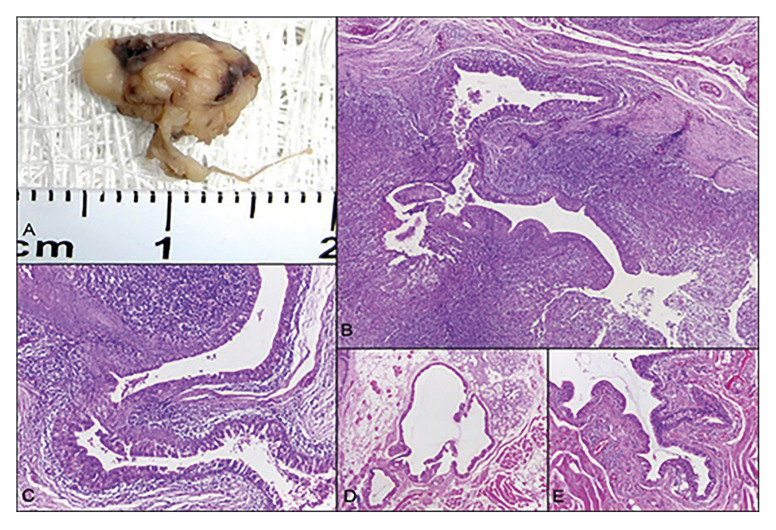
Macroscopic and microscopic aspects of cheilitis glandularis: (A) Gross examination showed a greyish and yellow nodule. (B-C) Ductal ectasia and salivary ducts with oncocytic metaplasia are associated with an intense inflammatory mononuclear infiltrate. (D-E) Minor salivary glands with ductal ectasia, and oncocyte metaplasia. Presence of mucin was observed. (hematoxylin and eosin stain; original magnifications: B - 40X, C, D, E - 100X).

Case 4: A 59-year-old female patient consulted relating multiple painless nodules on the lower lip mucosa. The patient was in good general health had no relevant medical history. She did not have lip trauma, smoking or alcohol use. Intraoral examination revealed multiple submucosal firm nodules, of normal coloration, being asymptomatic on palpation. Local excision of the tumors was performed. Microscopically, ductal ectasia of minor salivary glands with oncocyte metaplasia and the presence of mucin were seen (Fig. 3). The final diagnosis was CG. No recurrence was observed 5 years after treatment.

Case 5: A 67-year-old male patient was referred for painless lip inflammation of 8 months. He was a chronic alcohol drinker and a heavy smoker, presented with controlled hypertension, had a history of depression, and was taking anxiolytics without supervision. The patient also reported being diabetic with no treatment.

**Figure 4:**
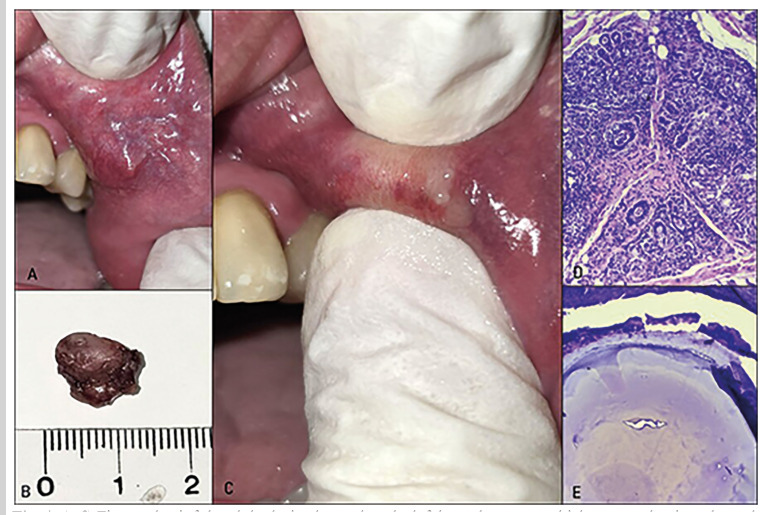
A-C. Firm and painful nodular lesion located on the left buccal mucosa, which, upon palpation, showed the discharge of a purulent secretion mixed with viscous saliva. B. Surgical specimen following complete resection. D. Ductal ectasia and intense periductal inflammatory infiltrate. E. Sialolith of the minor salivary gland in the context of SSC (hematoxylin and eosin stain; original magnifications: D - 10X, E - 40X).

Oral inspection showed two nodular lesions on the buccal mucosa on both sides. The lesions were not painful and on palpation, a thick saliva discharge mixed with pus was seen. Fetid halitosis, and periodontal disease were also identified. Blood glucose was 345 mg/dl, signifying a serious diabetes imbalance. Amoxicillin with clavulanic acid (875 mg/125 mg) plus metronidazole 250 mg was prescribed for two weeks. A biopsy of the left buccal mucosa revealed chronic sialadenitis, low periductal inflammation, and oncocytic metaplasia in the glandular ducts. CG was diagnosed.

Case 6: A 42-year-old male patient was referred due to painless swelling of 3 months' duration located on the left buccal mucosa. His medical history included depression, and he was taking anxiolytics without medical supervision. The patient also reported parafunctional habits, specifically self-biting of the buccal mucosa. Clinical examination revealed a firm, painful nodular mass on the buccal mucosa. A thick saliva discharge mixed with pus was observed. An excisional biopsy of the lesion demonstrated chronic sialadenitis characterized by periductal inflammation and oncocytic metaplasia within the glandular ducts. Additionally, a rounded calcified structure consistent with a minor salivary gland sialolith was identified. Based on these findings, a diagnosis of sialadenitis secondary to minor salivary gland sialolithiasis (SSG) was established (Fig. 4). The patient was treated with amoxicillin-clavulanic acid (875 mg/125 mg) combined with metronidazole (250 mg) for two weeks. Complete resolution of the lesion was observed following treatment.

## Results

The cases included in this study demonstrated a higher prevalence of this condition in males, with a male-to-female ratio of 1:2, and a mean age of 65.33 years (range: 42-78). Tobacco use was reported in 50% of the patients, with the majority being heavy smokers. Additionally, comorbidities or medications associated with xerostomia were identified in most cases, with depression and its related pharmacological treatments being the most common.

The most frequently recorded local factors associated with clinical lesions included poor periodontal health, the presence of dental septic foci, and chronic trauma due to self-inflicted injury. In all cases, the lesions presented as firm, painful nodules upon palpation, with a sessile, poorly defined, fluctuating base. The surface was smooth, with preserved mucosa, although small openings consistent with fistulas were observed. These fistulas discharged fluids resembling pus, viscous saliva with an egg white-like consistency, or a mixed fluid secretion.

## Discussion

CG was first described in 1870 as an oral condition that affects minor salivary glands of the lips. 100 years later, Williams and Williams published a case with the involvement of palatal salivary glands. They described clinical and histopathological features similar to CG, based on a dilated secretory duct with a dense chronic inflammatory infiltrate, acinar atrophy, oncocytic change and focal infiltrations of chronic inflammatory cells. However, when extralabial minor salivary glands are involved, this entity is mentioned as SSG (3). Regarding anatomical involvement, the current criteria for diagnosis of such entities are not clear enough to properly differentiate CG from SSG. SSG refers to a more generalized clinical scenario with the involvement of minor salivary glands located in the buccal, palatal, ventral tongue mucosa, etc. Cases comprising salivary glands of the buccal mucosa have been described as CG, but we suggest it is best to use this terminology only for exclusive labial mucosa involvement. This issue is relevant not only to establish a classification of the disease but also for a proper characterization of the associated etiological factors since CG does not necessarily share the same factors as SSG.

Clinical variants of CG include simple type, superficial suppurative, and deep suppurative types (8). Our cases were diagnosed in a clinical scenario of suppurative variants. In relation to the clinical manifestations, our cases were consistent with what is typically described in the literature (4,6,7), specifically the presence of firm nodules with erythematous dilated ostioles of the minor salivary glands, through which viscous, low-flow saliva, sometimes mixed with purulent exudate, is discharged. The lesions may or may not be painful, depending on their progression and the degree of involvement of the affected minor salivary gland. Reasonably, all lesions are located in areas where minor salivary glands are anatomically present. The most common sites are the lower labial mucosa and the buccal mucosa. However, one of the cases reported in this study exhibited multiple locations, including involvement of the palate's salivary glands, an anatomical site less frequently reported in the literature. The differential diagnosis of CG includes other clinical entities that may take place on lips, such as multiple mucocele, angioedema, minor salivary gland tumors, factitious cheilitis, chronic sialadenitis of the minor salivary glands, actinic cheilitis, and infectious diseases (7). When the lip is exclusively involved, the primary differential diagnoses for CG include irritant or allergic contact cheilitis, mechanical cheilitis, granulomatous cheilitis associated with Melkersson-Rosenthal syndrome, sialolithiasis, actinic cheilitis, and IgG4-related disease. Although the microscopic features of CG are nonspecific, the most common findings include mucus accumulation in the ductal lumen, ductal ectasia, and chronic sialadenitis (1,4). The absence of dense lymphocytic infiltrate, fibrosis, and obliterative phlebitis, which are the major histopathological features of IgG4-related disease, lack of any other glandular involvement and plasma cell infiltration, and detection of normal serum IgG4 level provided the exclusion of lip involvement of IgG4-related disease (8). The correct diagnose of CG and SSG needs a careful medical history, clinical examination, obtaining a deep biopsy of salivary glands. Therefore, histopathological findings must always be evaluated in conjunction with clinical characteristics to establish an accurate final diagnosis (7).

Although an autosomal dominant pattern of inheritance has been proposed, other factors have been suggested: syphilis (4), stress (9), wind and sunshine (9,10), smoking (5,10), and poor oral hygiene (5,10). Exogenous factors such as actinic damage could be relevant for cases where the lower lip is exclusively involved. Nevertheless, there may be other influences when sites not exposed to the aforementioned factors are found (such as the case of palatal gland involvement in SSG in case 1). Interestingly, although chronic exposure to actinic damage has been implicated as a causative factor of CG (9,10), none of the cases described here showed an association with climatic or actinic factors. However, they presented poor oral health leading to oral dysbiosis, mechanical irritation, heavy tobacco consumption, or medication-related hyposalivation. Therefore, the actinic damage could be considered an aggravating factor of cases of CG when the mucosa of the lower lip is exposed to UV radiation (6).

Hyposalivation is the objective reduction of salivary flow rate, usually defined as an unstimulated whole saliva flow rate of <0.1 mL/min, collected for 5 to 15 min (11). On the other hand, xerostomia is the subjective sensation of dry mouth (12). The sensation of dry mouth and associated factors, including chronic smoking, diabetes mellitus, and medication use, were documented in the majority of the cases included in the study. Consequently, both hyposalivation and xerostomia should be always recorded in patients with CG or SSG. Hyposalivation could produce an imbalance in aquaporins (AQPs). Nico *et al*. studied AQPs in patients with CG, finding differences in the expression of AQPs 1, 2 and 8, stating that their function is altered in CG. This could generate alterations in the final composition of saliva, making it more viscous than normal saliva (10). In addition, this could prompt a retrograde bacterial infection with suppuration (4). Medication-induced hyposalivation is one of the most common adverse effects of several drugs, including anxiolytics, antidepressants and antihypertensives, and others. Two of our cases were on antihypertensive treatment. Besides, four patients suffered from depression and also were using anxiolytics and antidepressants. These drugs were previously associated with the disease (13,14).

The diagnostic criteria suggested by Piperi *et al*. are based on clinical and histopathological features (4,6), with histopathological findings being almost always present. However, these diagnostic criteria could be refined by incorporating anatomical locations, allowing for a more precise differentiation of the affected salivary glands. Specifically, the diagnosis of CG should be restricted to cases involving only the labial salivary glands (upper, lower, or both). In contrast, cases where salivary glands in other oral cavity sites—such as the buccal or palatal regions—are affected could be categorized as SSG. Based on these criteria, cases 3 and 4 fulfill the diagnosis for CG, while cases 1, 2, 5, and 6 align with the diagnosis of SSG. Table 2 illustrates the Reiter criteria alongside our proposed adaptation. Including the anatomical location of the affected glands in the classification criteria for CG and SSG could help clinicians establish more accurate diagnoses, considering potential triggering factors and tailored treatment strategies. As described in this study, common factors between CG and SSG include hyposalivation or glandular alterations (due to emotional factors or medications that induce hyposalivation) (10), while aggravating factors differ. For CG, sun exposure and direct contact with infectious foci are significant, whereas mechanical irritation or smoking are more associated with SSG. This proposed classification, which considers the anatomical location of the affected glands—whether labial, extralabial, or mixed—could provide a more individualized approach to understanding the etiology of these conditions, ultimately improving therapeutic outcomes. Given the limited number of reported cases, it remains challenging to determine whether this classification could also predict lesion prognosis. The literature shows instances of recurrence in both conditions (CG and SSG), with therapeutic regimens often varying (7). Refining the diagnostic criteria by incorporating the anatomical location of the affected glands could enhance the accuracy of diagnosis for these rare and underreported conditions, facilitating a deeper understanding of their origins, contributing factors, and more personalized treatment approaches.

In four of our cases, in addition to the complete surgical removal of the affected gland, antibiotics were prescribed due to the high prevalence of periodontal disease in the context of diabetes and the presence of evident infection, as indicated by purulent discharge. This therapeutic approach was based on the available scientific evidence. In the systematic review by de Sales *et al*., 10 studies reported the use of antibiotics as part of the therapeutic arsenal. The most commonly prescribed antibiotics were cephalexin 500 mg (with no accepTable outcomes) and doxycycline 100 mg. In other instances, minocycline, known for its combined antimicrobial and anti-inflammatory properties, were used in combination with topical tacrolimus, which has proven to be an effective conservative alternative for managing CG (7-9). In our cases, we also selected for the use of amoxicillin and metronidazole due to its significant concentrations in saliva (15). Additionally, the consistent use of sunscreen is essential, as the affected region is already exposed to aggressive environmental factors in cases of CG. However, when conservative therapy fails to achieve long-term improvement, surgical removal of the affected minor salivary glands should be considered for patients with persistent symptoms. Despite the variety of management strategies described in the literature, no consensus or evidence-based guidelines have been established to definitively recommend the most appropriate treatment approach.

## Conclusions

Both CG and SSG are uncommon and challenging disorders of the oral cavity. Previous studies highlighted hyposalivation as an early phenomenon and its multifactorial nature, similar to our cases. In these patients, documenting factors contributing to xerostomia (such as comorbidities or associated medications) and addressing local sources of inflammation that contribute to disease development are crucial for a comprehensive management approach. Our proposed diagnostic criteria could help define CG and SSG more precisely. However, additional reports are necessary to gain a deeper understanding of these diseases.

## Figures and Tables

**Table 1:** Description of clinical cases.

Case	Gender	Age	Comorbidities	Tobacco consumption	Local factors	Medication	Affected salivary glands	Clinical criteria (by Reiter et al)	Histopathological criteria ( by Reiter et al)
#1	Male	78	Arterial hypertension Depression	Former smoker	Ill-fitting dentures Periodontal disease Poor hygiene Halitosis	Antihipertensives Clonazepam 0.5mg/day Sertraline 25mg	Buccal bilateral Palatal Upper Labial	Both clinical criteria	Chronic inflammation Mucous/oncocytic metaplasia (ducts and/or acini) Mucin in ducts
#2	Male	71	Chronic gastritis Depression	Heavy Smoker	Poor dental condition P eriodontal disease Halitosis	Clonazepam 0.5mg/day	Buccal Upper Labial Lower Labial	Both clinical criteria	Sialectasia Chronic inflammation Mucous/oncocytic metaplasia (ducts and/or acini) Mucin in ducts
#3	Female	75	Sgögren syndrome	No	None	No data	Lower Labial	Both clinical criteria	Chronic inflammation Mucous/oncocytic metaplasia (ducts and/or acini) Mucin in ducts
#4	Female	59	No	No	None	None	Lower Labial	Both clinical criteria	Sialectasia Chronic inflammation Mucous/oncocytic metaplasia (ducts and/or acini) Mucin in ducts
#5	Male	67	Chronic alcoholic Arterial hypertension Depression Type II Diabetes Mellitus	Heavy smoker	Poor dental condition Periodontal disease Halitosis	Clonazepam 0.5mg/day	Left and right buccal mucosa	Both clinical criteria	Chronic inflammation Mucous/oncocytic metaplasia (ducts and/or acini)
#6	Male	42	Depression Anxiety	No	Chronic Trauma	Clonazepam 0.5mg/day	Left buccal mucosa	Boch clinical criteria	Sialectasia Chronic inflammation Mucin in ducts Syalolith

**Table 2:** Proposed criteria for CG and SSG diagnosis including the involved anatomical area.

	Clinical diagnostic Criteria (A)	Histopathological diagnostic criteria (B)
Reiter criteria for CG	a)Multiple lesions: involvement of more than one minor salivary gland b)Mucoid and/or purulent discharge from the apertures of the involved minor salivary glands.	a) Sialectasia b) Chronic inflammation c) Mucous/oncocytic metaplasia (ducts and/or acini) d) Mucin in ducts
	A Both mandatory B ≥2 criteria must be present	
Our criteria for CG and SSG	a) Multiple lesions: involvement of more than one minor salivary gland. 1.1 Cheillitis Glandularis; involvement of one or more salivary glands of labial location (upper or lower). 1.2 Stomatitis Glandularis; involvement of one or more salivary glands of labial and extralabial location, or extralabial involvement. b) Mucoid and/or purulent discharge (suppuration) from the involved minor salivary glands.	a) Sialectasia b) Chronic inflammation c) Mucous/oncocytic metaplasia (ducts and/or acini) d) Mucin in ducts

A. Clinical criteria must be established to differentiate the anatomical location of the affected minor salivary glands. Involvement of only the labial salivary glands supports the diagnosis of CG, whereas involvement of one or more extralabial salivary glands suggests a diagnosis of SSG. b) This clinical phenomenon must be present as an indicator of the suppurative nature of the disease. B. Histopathological diagnostic criteria. ≥2 histological findings must be present. A and B criteria should be met for the diagnosis.
